# MLACNN: an attention mechanism-based CNN architecture for predicting genome-wide DNA methylation

**DOI:** 10.1007/s12064-023-00402-3

**Published:** 2023-08-30

**Authors:** JianGuo Bai, Hai Yang, ChangDe Wu

**Affiliations:** Shandong Jiaotong University, Jinan City, Shandong Province China

**Keywords:** Genome wide methylation detection, Attention CNN, Hybrid neural network

## Abstract

Methylation is an important epigenetic regulation of methylation genes that plays a crucial role in regulating biological processes. While traditional methods for detecting methylation in biological experiments are constantly improving, the development of artificial intelligence has led to the emergence of deep learning and machine learning methods as a new trend. However, traditional machine learning-based methods rely heavily on manual feature extraction, and most deep learning methods for studying methylation extract fewer features due to their simple network structures. To address this, we propose a bottomneck network based on an attention mechanism and use new methods to ensure that the deep network can learn more effective features while minimizing overfitting. This approach enables the model to learn more features from nucleotide sequences and make better predictions of methylation. The model uses three coding methods to encode the original DNA sequence and then applies feature fusion based on attention mechanisms to obtain the best fusion method. Our results demonstrate that MLACNN outperforms previous methods and achieves more satisfactory performance.

## Introduction

Epigenetic modification is a essential modification which can reversible and heritable regulate gene function without nuclear DNA sequence change. At present, the known epigenetic phenomena mainly include DNA methylation, genomic imprinting, maternal effects, gene silencing, nucleolar dominance, dormant transposon activation and RNA editing (Jones 2012; Robertson 2005; Suzuki and Bird 2008; Nye et al. 2020; Cheng et al. 2018). DNA methylation traditionally refers to 5mC, but some new DNA methylation modifications have been found recently, such as 6mA, 4mc (Ma et al. 2014; O’Brown and Greer 2016; Yu et al. 2015). DNA methylation at the cytosine (5mC) 5 position in the genome is a major and common epigenetic event to various cellular processes among all types of DNA methylation.

Predicting the methylation level of methylation sites is an important area of research in epigenetics. With the rapid development of sequencing technology, various methods have been developed to detect DNA methylation levels. The gold standard for genome-wide methylation determination is WGBS (Habibi 2013) (whole-genome bisulfite sequencing), which delivers high accuracy and repeatability while covering the methylation status of every C base in the genome. ScWGBS (Huang 2019) (single-cell whole-genome bisulfite sequencing) is a WGBS method designed for analyzing the methylation state of every cytosine in a single cell with high accuracy. However, because of its high cost, researchers have developed cheaper alternative detection methods. Although RRBS (Wang et al. 2013) (reduced representation bisulfite sequencing) significantly reduces the amount of sequencing needed and enables comparative genome analysis of multiple samples, it introduces new limitations: limited digestion sites and inefficient digestion efficiency that reduce the amount of sequencing information obtained. MeDIP (Jacinto et al. 2008) (methylated DNA immunoprecipitation sequencing), which detects genome-wide methylation with less sequence data, has lower accuracy than WGBS and may produce erroneous results due to the use of specifically bound antibodies (Petterson et al. 2014). RRHP (reduced representation of 5-hydroxymethylcytosine profile) can detect 5hmc in the genome, but only in specific enzyme digestion sites. In comparison, oxBS-Seq (Booth et al. 2012) (oxidative bisulfite sequencing) can distinguish between 5mC and 5hmc, although it requires special reagents that increase the cost and time of methylation analysis. The choice of detection technology ultimately depends on research goals and available resources.

Compared to physical and chemical experiments for detecting methylation sites, machine learning and deep learning methods are undoubtedly simpler, more convenient, and cost-effective. Whether predicting methylation sites using machine learning or deep learning methods, they mainly use one or more of the following coding methods: k-mer, one-hot, ANF (accumulated nucleotide frequency), CKSNAP (composition of *k*-space nuclear acid pairs), DNC (dinucleotide composition), ENAC (enhanced nucleic acid composition), NAC (nucleic acid composition), RCKmer (reverse composition KMER), TNC (trinucleotide composition), EIIPs (electron-ion interaction pseudopotentials) of trinucleotides, NCP (nucleotide chemical property), and PseDNC (pseudo dinucleotide composition). Currently, machine learning methods are primarily combined with SVM (support vector machine) (Basith et al. 2019; Chen et al. 2015a, b, c, 2017a, b, 2019; Feng et al. 2019; Xiang et al. 2016; Xing et al. 2017; Akbar and Hayat 2018), Markov model (Pian et al. 2021; Yang et al. 2020), KNN (k-nearestneighbor) (Jia et al. 2016), RF (random forest) (Zhou et al. 2016; Manavalan et al. 2019) and XGBoosting(extreme gradient boosting) (Qiang et al. 2018; Liu and Chen 2020) to predict methylation sites through one or more coding methods mentioned above. RF and XGBoosting algorithms are mainly used to predict by integrating several weak classifiers such as SVM, Markov model and KNN. Traditional machine learning methods depend heavily on manual feature selection based on prior knowledge, and feature selection is crucial to the classification results. However, deep learning obviates the need for complicated manual feature selection and can automatically learn and classify complex methylation features through a series of network layers, thus simplifying the process and improving its accuracy.

Tang et al. (2020). proposed the INTERACT model, which utilizes a combination of convolutional neural networks and the Transformer model to predict the impact of genetic variation on DNA methylation levels at CpG sites in the human brain. This approach overcomes the challenges associated with identifying causal genetic variations that drive DNA methylation levels due to extensive linkage disequilibrium in the genome. PretiMeth (Zhou et al. 2022) is a novel method for constructing precise prediction models for DNA methylation at single CpG loci. The study demonstrates that the method accurately predicts DNA methylation levels and identifies several CpG loci and genes that are differentially methylated between tumor and normal samples, highlighting its potential for biological validation and expanding methylation array data. Angermueller et al. (2017) proposed DeepCpG. The method of feature fusion based on RNN and CNN is used to predict the two feature extraction methods for the input methylation data through simple feature splicing. RNN extracts the temporal features of methylation data, and CNN extracts the local sequence features of methylation. The network used is relatively simple, so the extracted features are relatively limited. Nazari et al. proposed iN6 Metal (5 steps) (Nazari et al. 2019), which extracts k-mer features of sequences and puts them into word2vec for feature selection, and finally uses CNN to classify features and predict. MRCNN (Tian et al. 2019) encodes the DNA sequence using one dot encoding method, and obtains 400 through a 1 * 4 one-dimensional convolution $\times$ The vector of 1 is folded to form 20 × 20 matrix, and then get the output after three layers of convolution. The network used is also relatively simple, and the features extracted are relatively limited. Moreover, simple folding of data may destroy the original structure of data.

Most of the previous deep learning methods for predicting methylation (Ma et al. 2020; Fu et al. 2021; Abbas et al. 2020; Rehman et al. 2021; Xu et al. 2021; Zhang et al. 2015; Zeng and Gifford 2017; Alam et al. 2020; Liu et al. 2021) used neural networks with insufficient depth or processed neural network inputs based on prior knowledge, resulting in limited effective features being extracted. Here, we propose MLACNN for predicting DNA methylation at site resolution. MLACNN encodes methylated sequences in three coding methods: one-hot, NCP and EIIP-vector coding. The encoded sequences are input into three RNN neural networks with attention mechanism and feature fusion based on attention mechanism is performed to obtain the predicted results of methylation. In our 20 experiments, the system has a median validation accuracy of 97.9% and an independent test accuracy of 94.8%. It is a relatively robust methylation prediction system with good prediction performance for both negative and positive samples. MLACNN is provided as an open-source tool available at https://github.com/jrebai/MLACNN.

## Methods

### data preprocessing

We used WGBS DNA methylation data: The original data were obtained from GEO (Gene Expression Omnibus database, https://www.ncbi.nlm.nih.gov/geo). The database downloaded the m5C methylation data with the accession number of gsm432685 (whole-genome shotgun bisulfite sequencing of the H1 cell line) and the mapped GRCh37 (genome reference consor Tim human reference 37) data.

The methylation sites and methylation prediction values are reserved for the methylation data downloaded from GEO; and then, the processed data are mapped with GRCh37 to obtain sequence methylation data of up and down 400 bp around the methylation sites. Sequence methylation data were encoded by one-hot, NCP and EIIP-vector as inputs to the neural network. Wherein the one-hot coding sequence methylation data is encoded as:

1 $$\begin{aligned} S = (s_{1},s_{2},\ldots , s_{n} ),~~s_{i}\in {\left\{ \begin{array}{ll} \text {A }:(1,0,0,0)^\textrm{T} \\ \text {T }: (0,1,0,0)^\textrm{T}\\ \text {C }:(0,0,1,0)^\textrm{T}\\ \text {G }:(0,0,0,1)^\textrm{T} \end{array}\right. },~i\in [1,n] \end{aligned}$$

The NCP coding method is based on the chemical properties of the four bases. By judging whether the four bases contain cyclic structures, functional groups and hydrogen bonds, the obtained DNA sequences are coded as follows:

2 $$\begin{aligned} S = (s_{1},s_{2},\ldots , s_{n} ),~~s_{i}\in {\left\{ \begin{array}{ll} \text {A }:(1,1,1)^\textrm{T} \\ \text {T }: (0,0,1)^\textrm{T}\\ \text {C }:(0,1,0)^\textrm{T}\\ \text {G }:(1,0,0)^\textrm{T} \end{array}\right. },~i\in [1,n] \end{aligned}$$

The EIIP-vector coding method is based on the chemical properties of four bases. By judging the four bases, the energy of delocalized electrons in nucleotides, the four bases A, T, C and G are coded as follows:

3 $$\begin{aligned} S = (s_{1},s_{2},\ldots ,s_{n} ),~~s_{i}\in {\left\{ \begin{array}{ll} \text {A }:(15.27,0,0,0)^\textrm{T} \\ \text {T }: (0,31.61,0,0)^\textrm{T}\\ \text {C };(0,0,-77.48,0)^\textrm{T}\\ \text {G }:(0,0,0,30.59)^\textrm{T} \end{array}\right. },~i\in [1,n] \end{aligned}$$

**Fig. 1:**
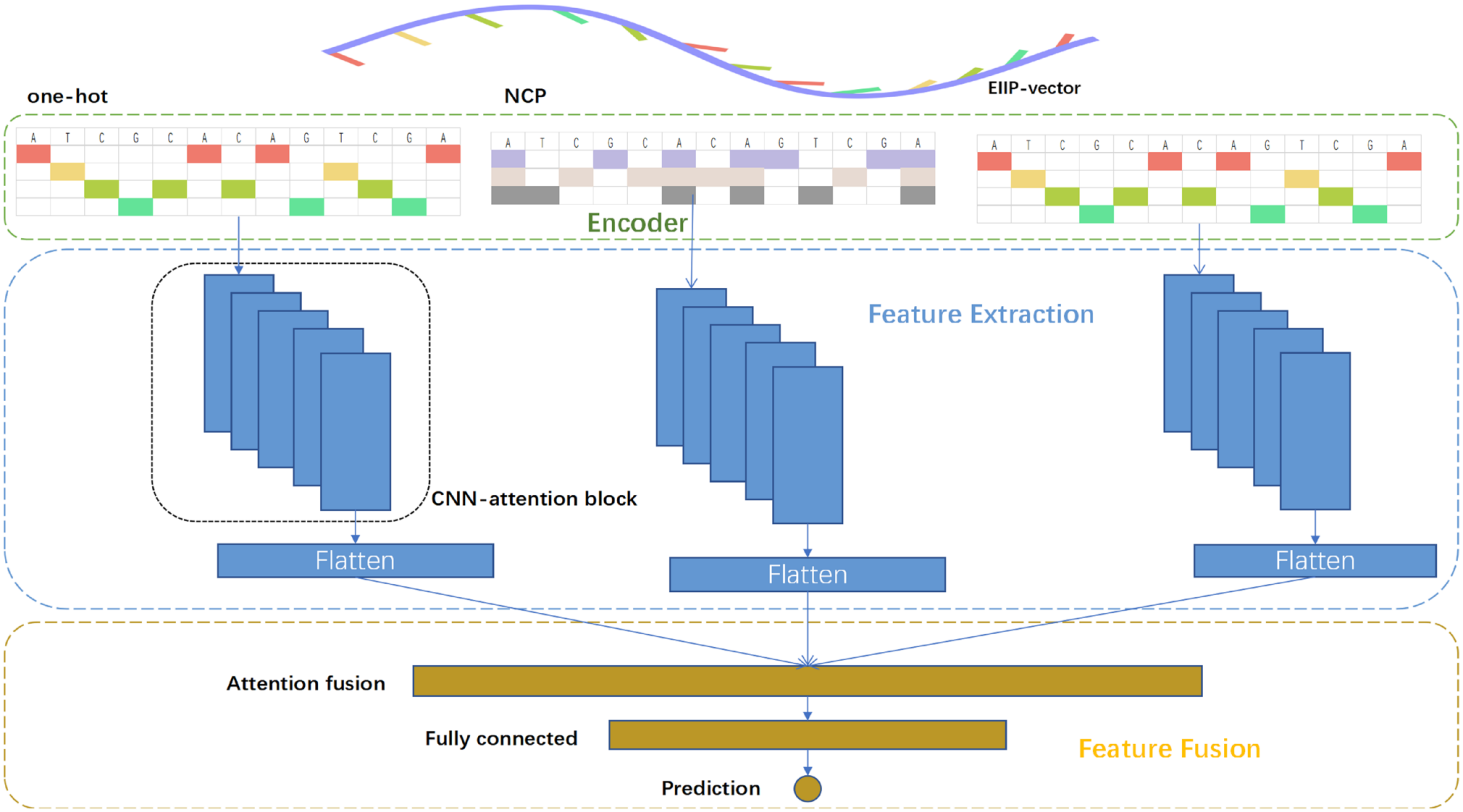
Abstract modular structure of MLACNN: The original data enter three similar feature extraction modules through three coding methods to obtain abstract features; and then, features are fused based on attention mechanism in feature fusion module to obtain prediction results

### Attention and bottleneck

Mnih et al. (2014) proposed a method that can extract information from a picture or a video, select a series of regions or locations through the supervisor, and only process the selected regions in a high-resolution way. Since then, attention mechanism has attracted extensive attention and research. Bahdanau et al. (2014) introduced attention mechanism into natural language processing for the first time. They performed translation and alignment simultaneously in machine translation tasks. Their model is superior to traditional encoder and decoder methods in English French translation tasks. Vaswani et al. (2017) proposed an attention mechanism transformer that is still widely studied until now. Its input is word embedding with location information. Through the encoder and decoder based on multihead self-attention, the correlation between words is extracted and sentence translation is made. Later, a series of soft attention mechanisms were put forward: spatial attention (Zhu et al. 2019), SENet (Sequence and Exception Net) (Hu et al. 2018), ECA-Net (Wang et al. 2020), etc. The soft attention mechanism automatically assigned larger features to important features by dynamically learning different weights of channels or spaces to obtain better classification results. We use soft attention to train our neural network by applying different attention to channels and spaces.

In order to overcome the side effects of group convolution, Zhang et al. (2018) proposed a new channel shuffling operation to help information interact between characteristic channels and found that it can improve the prediction performance and obtain faster processing speed. As an important improvement of neural network, residual network (He et al. 2016) firstly applies the idea of jump connection to neural network, and based on jump connection, it can greatly reduce the problems caused by gradient disappearance and over fitting. He trained the neural network to more than 100 layers for the first time, and the obvious experimental results in his paper showed that if the structural design of the neural network is not good, then the performance of the shallow neural network is far worse than that of the shallow neural network. I think this may also be the reason why other researches on methylation only use one or two layers of neural networks. Therefore, we creatively introduce residual structure design to our neural network to predict methylation sites. As far as we know, the deeper the neural network is, the more features can be extracted. The building block and bottomline proposed in his paper can obviously achieve fewer parameters with fewer parameters (that is, smaller models) and obtain better prediction results.

**Fig. 2:**
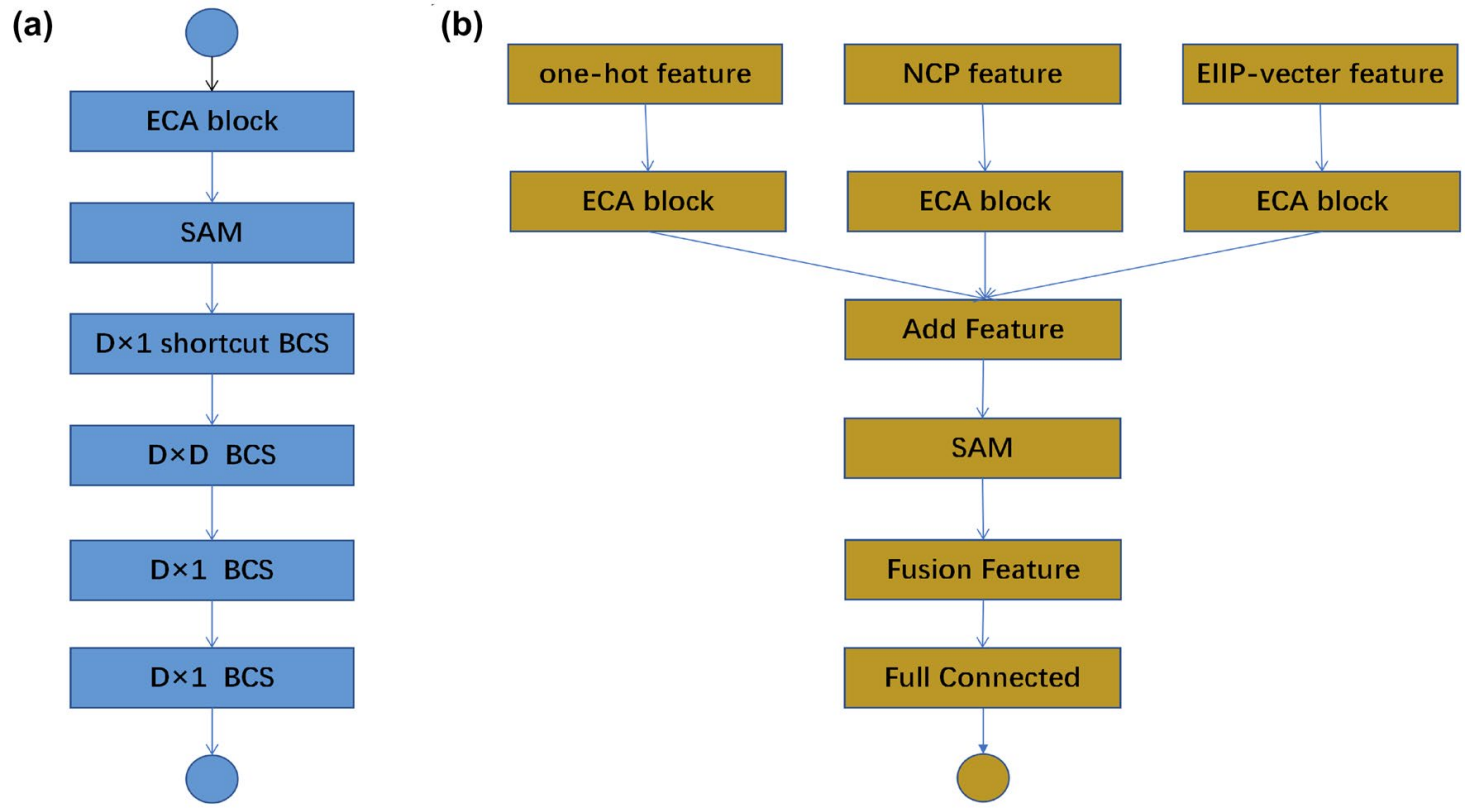
**a** Specific architecture of CNN attention block of MLACNN feature extraction module **b** specific architecture of feature fusion module

### Model

Most existing deep learning-based methods for predicting methylation sites suffer from two main shortcomings: shallow network layers that fail to extract enough high-dimensional features, resulting in underutilization of methylation data; and simple feature splicing or ensemble learning methods used when multiple coding methods or network architectures are employed to jointly predict methylation sites, leading to insufficient utilization of extracted features. To address these issues, we propose a model based on feature fusion and attentioned deep CNN. Our model consists of three processes: Encoder, Feature Extraction, and Feature Fusion, as shown in Fig. 1. The Encoder encodes the acquired methylation data to become the input of the neural network, the Feature Extraction extracts high-level abstract features of methylation, and the Feature Fusion fuses the learned high-dimensional features to obtain the prediction results through full connection network interaction. Compared to traditional neural network methods for predicting methylation sites, we use the shuffle channel method to disrupt the channels and enable each channel of a large-scale neural network to communicate more information and learn better features. We also employ ECA attention and Spatial attention methods to make the neural network training more successful and the learned features more focused on key information. For the fusion of multiple features, we use a special feature fusion method based on the attention mechanism. The features extracted from each network are first processed by ECA attention before feature fusion; and then, the fused features are SAM processed to make the fused features more focused on key information.

The MLA-BCS (multi-latitude attention bottle with channel shuffle) is the primary feature extraction component in our model, as we avoid using a simple stacked convolution layer to extract methylation sequence features due to the risk of gradient disappearance, overfitting, and ineffective feature extraction. By carefully stacking MLA-BCS modules, we can avoid the gradient disappearance phenomenon and ensure that the accuracy and other indicators of each training are relatively stable. The MLA-BCS first passes the input features through an ECA block and SAM to focus on channel and spatial features that are more conducive to identifying methylation sites. It then passes through BCS with convolution residual edges of $D\times 1$, BCS of $D\times D$, and BCS of two $D \times 1$, where *D* is the dimension of feature coding. Although BCS with convoluted edges can learn better features, it is more prone to overfitting than residual edges without convolution. Therefore, we use two convolutions without residual edges, and the purpose of using two-dimensional convolution is to enable interaction between features not only in the line direction. Our neural network uses four MLA-BCS to extract high-level convolution features, but the normal BCS module for convolution extraction of low-level features is also crucial: we first use a $5\times 1$ convolution kernel, then three $5\times 1$ to extract low-level features. For the feature fusion layer, we do not directly add or splice the extracted high-level features. Instead, we first focus on the channels of each feature through ECA block, then focus on the merged features, and then use the SAM module to make the fused features focus on the features after fusion. The SAM attention mechanism employs two convolutional layers, one for channel attention and the other for spatial attention, to learn spatial attention. During the learning process, a sigmoid function is used to limit the output range from 0 to 1, ensuring that the output is an effective attention weight. Unlike previous feature fusion methods, the SAM attention mechanism uses an attention mechanism for feature fusion. By learning different weights for different features, it assigns higher attention to more important features. Finally, we obtain the final prediction result by connecting the features after fusion through the full connection layer of 128 hidden neurons and then connecting with the output layer. Our model is also easy to disassemble and combine, as adding a new coding method for feature fusion only requires retraining a feature extraction and using the abstract features extracted by the feature fusion layer and other trained modules for fusion.

Specifically, we begin with a single strand of DNA with a length of *L* and use one-dot coding, NCP coding, and EIIP vector coding to form an $L \times D$ matrix, where *D* is the dimension of a single base. Our model employs the RMSprop optimizer to adjust the learning rate of each batch with a learning rate of 0.0001 and a batch size of 128. The cross-entropy function serves as the loss function of the neural network. To prevent overfitting, we use a combination of *L*1 and *L*2 regular functions to correct the weight and variance of the filter. Additionally, we employ early stopping to prevent overfitting in the epoch dimension. To further prevent overfitting, we utilize the max pooling method, which not only speeds up the fitting of neural networks but also prevents the network from being too deep and having too many parameters. For each pooling layer, we use a max pooling of $2\times 1$.

4 $$\begin{aligned} Y = \max \begin{Bmatrix} x_{1}&x_{2} \end{Bmatrix} \end{aligned}$$

where *Y* is the output of the current receptive field and $X_{i,j}$ is the current receptive field value

**Fig. 3:**
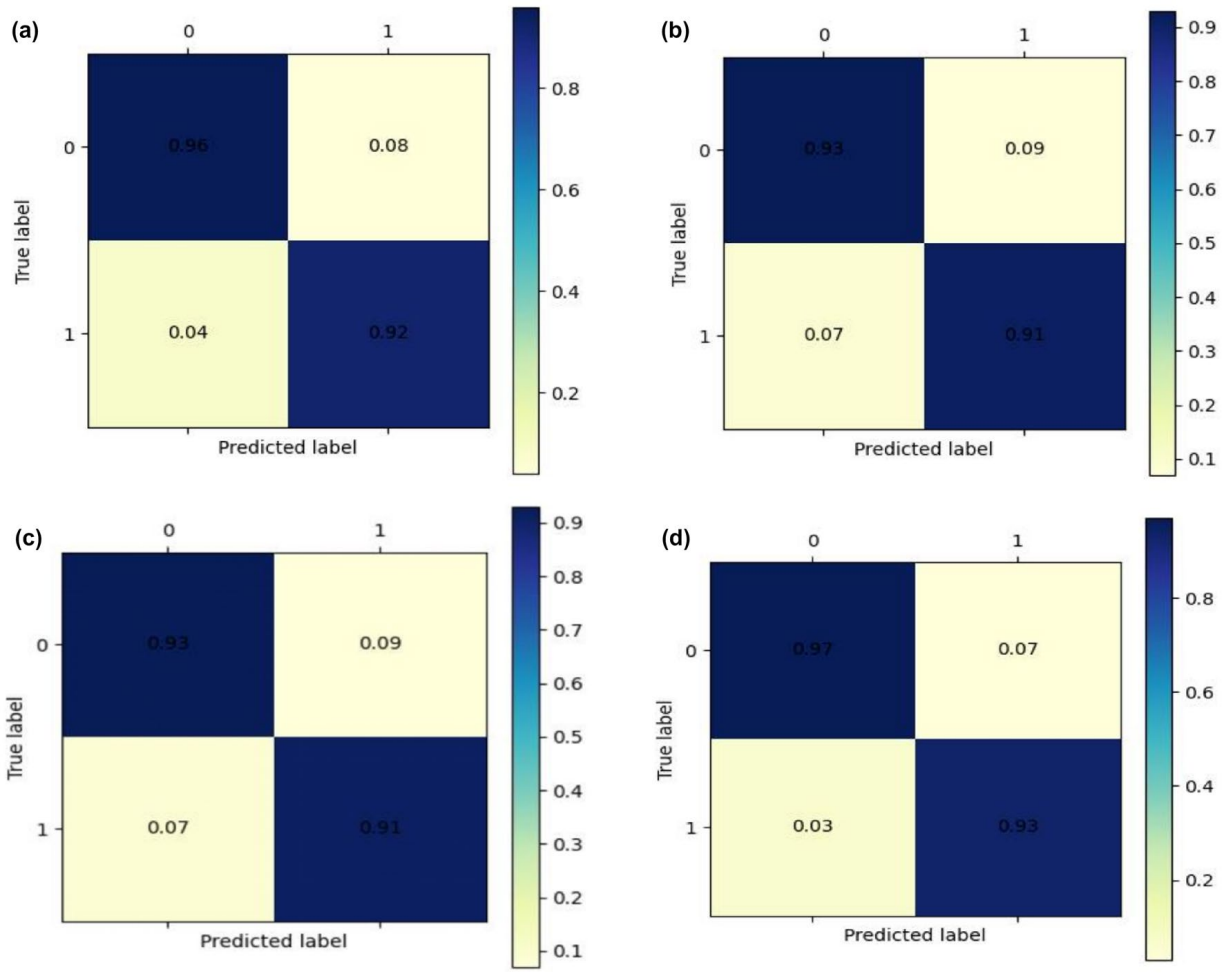
Confusion matrix of proposed model. Among them, **a**–**c** are the confusion matrices that use one pot encoding, NCP encoding, and EIIP-vector data processing methods to stack and use our MLA-BCS module to predict test set data. **d** For fusion, the features extracted in **a**–**c** are fused and the confusion matrix displayed on the test set is retrained

To prevent overfitting, we also added BN layer (Ioffe and Szegedy 2015) (batch normalization) after each conv2d convolution layer. The BN layer forcibly pulls the distribution of the input value of any neuron of the neural network of each layer back to the standard normal distribution with the mean value of 0 and the variance of 1 through certain normalization means, so that the activation input value falls in the area where the nonlinear function is sensitive to the input. In this way, the small change of the input will lead to the large change of the loss function, which means that the gradient will become larger and the problem of gradient disappearance will be avoided. And the larger the gradient means that the learning convergence speed is faster, which can greatly speed up the training speed. We also used Dropout (Srivastava et al. 2014) technology to randomly discard some neurons in the network to prevent overfitting.

We use the ReLu function (corrected linear activation function) for all activation functions except the final prediction result. For each output *Y*, the input *X* action function is as follows:

5 $$\begin{aligned} Y = {\text {ReLu}}(x) = {\left\{ \begin{array}{ll} \text {x }, \qquad x>0 \\ \text {0 }, \quad {\text {else}} \end{array}\right. } \end{aligned}$$

The activation function for the predicted value is sigmoid, which can ensure that the predicted value is between 0 and 1:

6 $$\begin{aligned} Y = {\text {sigmoid}}(x) = \frac{1}{1 + e^{-x}} \end{aligned}$$

The basic operation for convolution is $Y = {\text {Conv}} (X)$:

7 $$\begin{aligned} Y_{p,q} = {\text {Conv}}(X) = {\textstyle \sum \limits _{i=1}^{w_{c}}} {\textstyle \sum \limits _{j=1}^{D}}W_{q,i,j}X_{p+i,j}+b \end{aligned}$$

where **W** is the weight matrix of the convolution kernel, **X** is the input matrix of the current convolution kernel, and **b** is the offset of the convolution function

We will use the formula to represent the module in Fig. 2b as follows:

8 $$\begin{aligned} Y = {\text {fusion}}(A,B,C) = W\bullet {\text {SAM}}\begin{pmatrix} {\text {ECA}}(A)\\ {\text {ECA}}(B) \\ {\text {ECA}}(C) \end{pmatrix} +d \end{aligned}$$

We use Binary Cross Electron as our loss function:

9 $$\begin{aligned} H_{p}(q)= & {} 7 - \frac{1}{N}\sum _{i=1}^{N} y_{i}\bullet \log (p(y_{i})) + (1-y_{i})\nonumber \\{} & {} \quad \bullet \log (1-p(y_{i})) \end{aligned}$$

## Performance evaluation

**Fig. 4:**
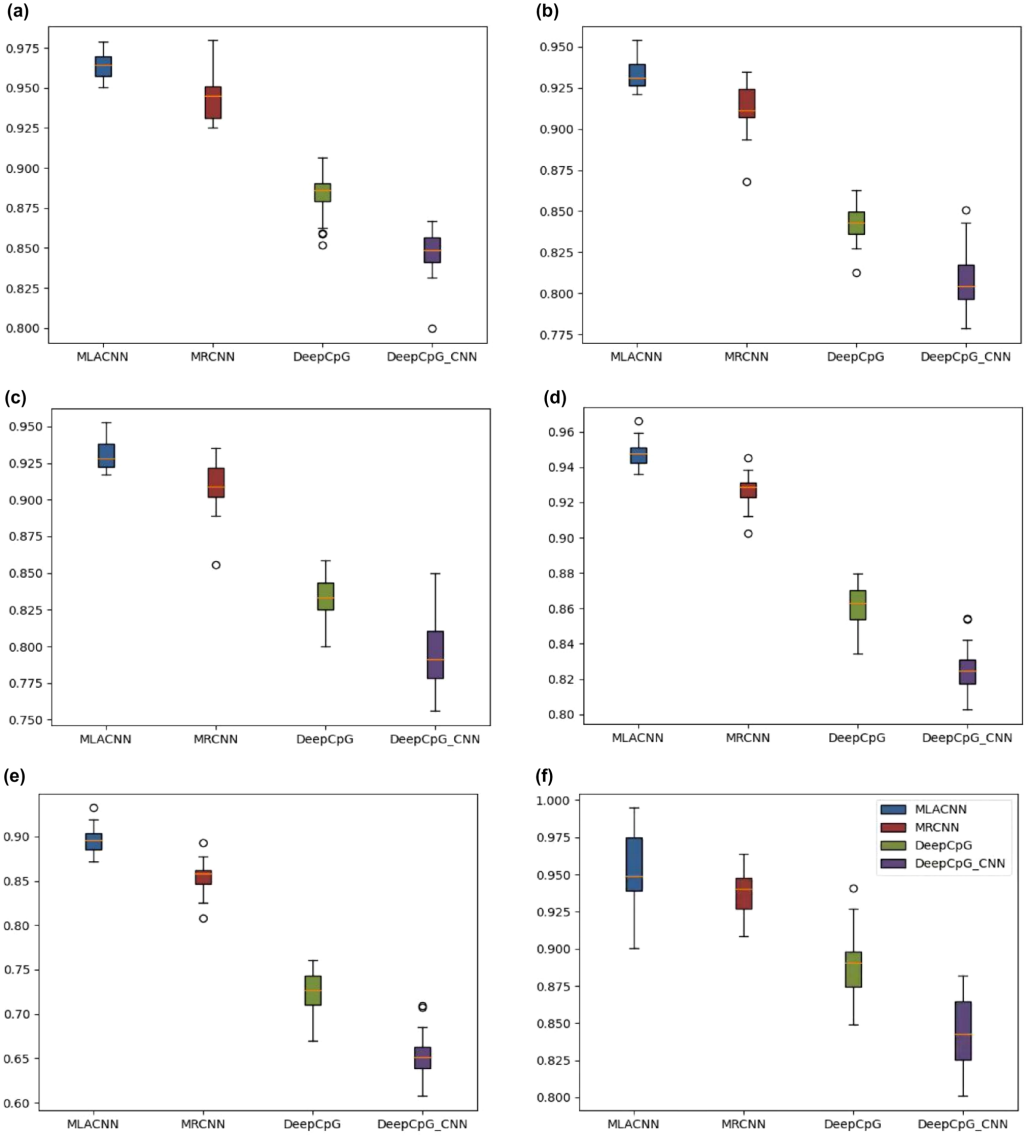
Boxplot of six indicators on MLACNN, MRCNN, DeepCpG and DeepCpG CNN: **a** Sn, **b** SP, **c** precision, **d** ACC, **e** MCC, **f** AUC

**Fig. 5:**
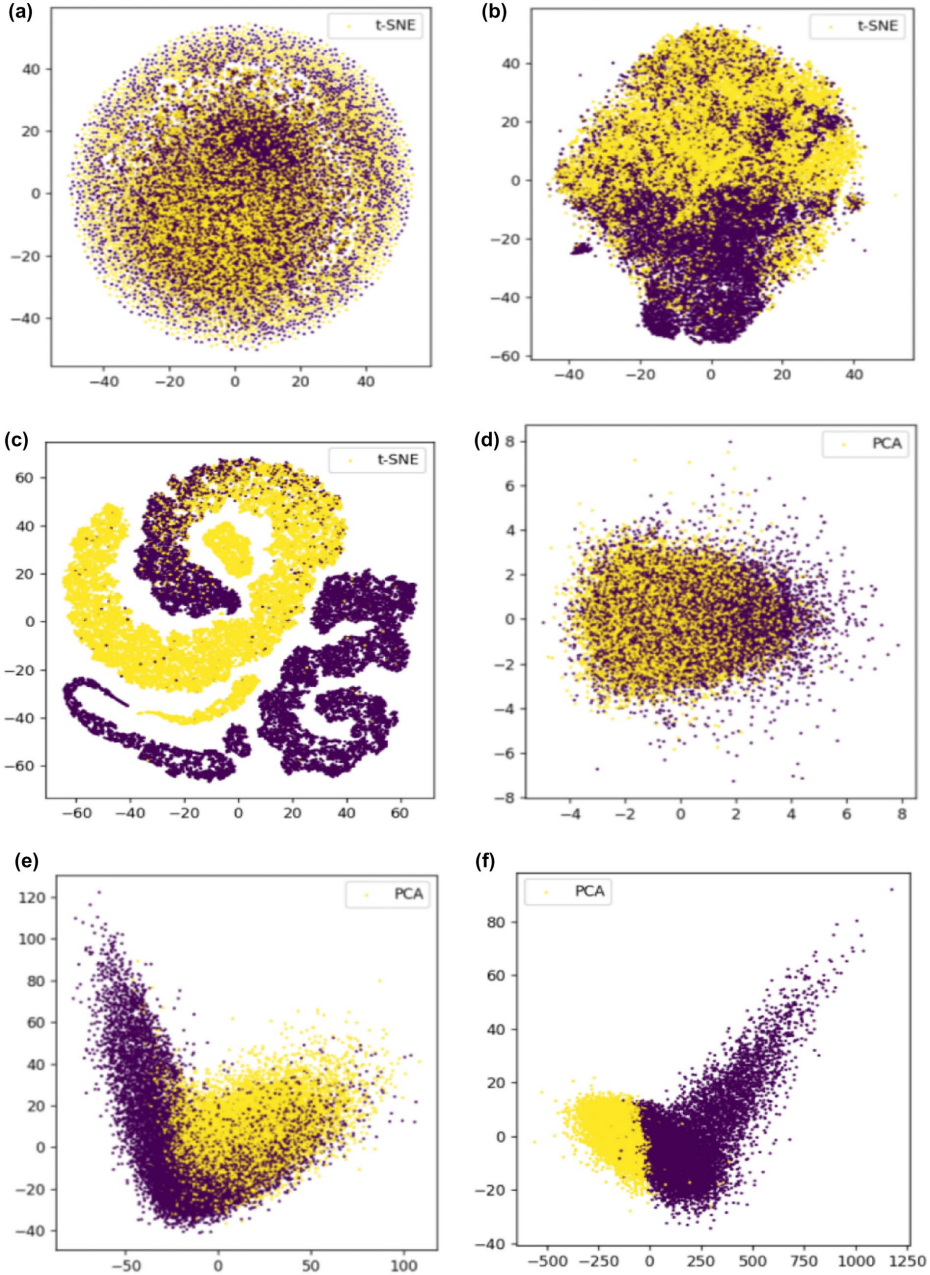
**a**–**c** Represent the visualization results of t-SNE clustering for raw data, data processed by the first layer of MLACNN, and data processed by the last fully connected layer, respectively. On the other hand, **d**–**f** show the visualization results processed by PCA

In order to quantify the performance of MLACNN and compare it with other methods, we used six common performance evaluation indicators: sensitivity (SN), specificity (SP), precision, accuracy (ACC), Matthew correlation coefficient (MCC), and AUC(area under the curve, refers to the area under the ROC curve).

10 $$\begin{aligned} Sn= & {} \frac{{\text {TP}}}{{\text {TP}}+{\text {FN}}} \end{aligned}$$

11 $$\begin{aligned} Sp= & {} \frac{{\text {TN}}}{{\text {TN}}+{\text {FP}}} \end{aligned}$$

12 $$\begin{aligned} {\text {Precision}}= & {} \frac{{\text {TP}}}{{\text {TP}}+{\text {FP}}} \end{aligned}$$

13 $$\begin{aligned} {\text {ACC}}= & {} \frac{{\text {TP}}+{\text {TN}}}{{\text {TP}}+{\text {FP}}+{\text {TN}}+{\text {FN}}} \end{aligned}$$

14 $$\begin{aligned} {\text {MCC}}= & {} \frac{{\text {TP}}\times {\text {TN}}+{\text {FP}}\times {\text {FN}}}{\sqrt{({\text {TP}}+{\text {FP}})\times ({\text {TP}}+{\text {FN}})\times ({\text {TN}}+{\text {FP}})\times ({\text {TN}}+{\text {FN}})} } \end{aligned}$$

where TP, FP, TN, and FN represent the number of true positives, false positives, true negatives, and false negatives, respectively (Figs. 4, 5).

s

## Results and discussion

Samples with a methylation $\beta$ value greater than 0.5 were set as positive samples, and samples with a methylation $\beta$ value less than or equal to 0.5 were set as negative samples. We first train and verify the same training set and test set for our model using one-hot coding method, NCP coding method and EIIP vector, and then train and verify the feature layer formed by the two coding methods based on the feature fusion model of attention mechanism, then obtain four models. The confusion matrix predicted by the four models is shown in Fig. 3. For the overall performance of the four models, compared with the other three coding models, their SP has been significantly improved, which can show that our fusion model has relatively good recognition ability for negative samples and is easier to avoid false positives. The confusion matrix predicted by the four models is shown in Fig. 3. It can be seen that better recognition effect can be obtained after feature fusion, which can reach 0.97 true positive and 0.93 true negative.

We compared our model with DeepCpG, DeepCpG RNN module and MRCNN. We tested the sensitivity (SN), specificity (SP), precision, accuracy (ACC), Matthews correlation coefficient (MCC) and AUC to compare the advantages and disadvantages of these three models. Among them, DeepCpG mainly uses one-hot coding and location information to input data into CNN and RNN models, and then performs simple feature fusion on the two coding methods to obtain results. The network structure used is relatively simple, so the extracted features are relatively limited. MRCNN uses a method similar to DeepCpG to one-hot code DNA data to obtain a $400\times 4$ matrix. After one-dimensional convolution, a $400\times 1$ vector is obtained, and then folded to form a $20 \times 20$ matrix. After three-layer convolution and a full connected layer, the output is obtained. The network structure used is relatively simple, and the extracted features are relatively limited. Moreover, simple folding of the data may damage the original structure of the data. Our model mainly uses one-hot, NCP and EIIP-vector coding methods to extract more high-level features through deeper convolution layer with attention mechanism, and then perform feature fusion based on attention mechanism to effectively predict methylation status. Comparing MLACNN with MRCNN, DeepCpG and DeepCpG CNN through six indicators, we can see that our model has significantly improved in various indicators. The median values of SN, SP, precision, ACC, MCC and AUC are 0.964253, 0.93088502, 0.92837567, 0.947527445, 0.89562882 and 0.948907645, respectively.

**Fig. 6:**
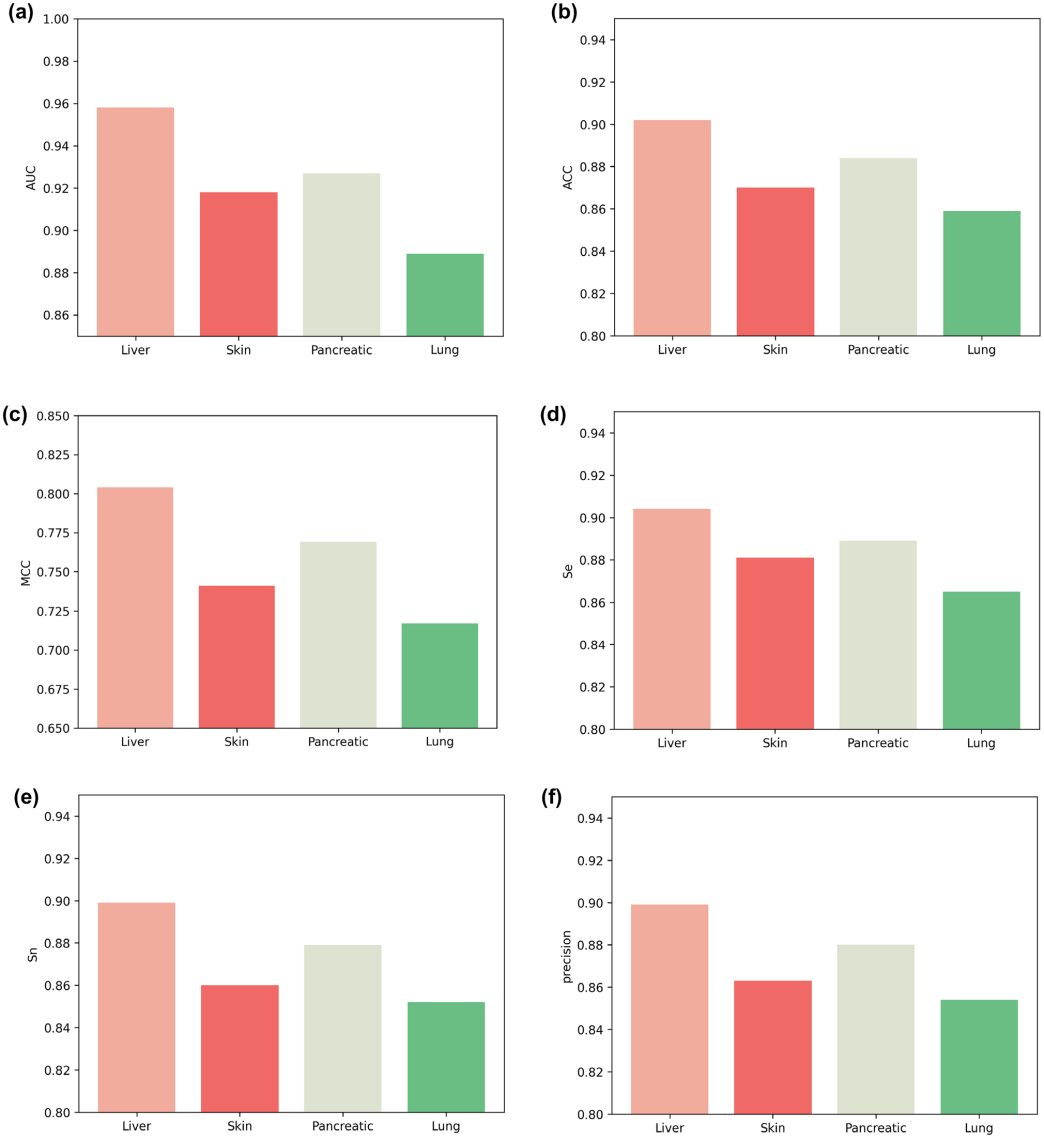
Six indicators of liver, skin, pancreatic, and lung cells on MLACNN: **a** AUC, **b** ACC, **c** MCC, **d** Se, **e** Sn, and **f** precision

We also evaluated the performance of MLACNN on different tissues and cell types. Specifically, we compared and predicted the performance of four tissue methylation levels, namely human liver tissue, human skin tissue, human pancreatic islet cells, and human lung tissue. The results are presented in Fig. 6, revealing that the ACC and AUC of MLACNN on human liver tissue are 0.904 and 0.958, respectively, while the worst performing human skin tissue still achieves an ACC and AUC of 0.859 and 0.889, respectively. Despite being based on human stem cell training, MRCNN demonstrates excellent performance on methylation data from other tissues, thus highlighting its effectiveness as a universal predictive tool for whole-genome methylation.

To explore the extraction and abstraction of DNA sequence information by CNN and attention mechanism, we conducted t-SNE (t-distributed stochastic neighbor embedding) and PCA (principal components analysis) operations on the raw sequence without any processing, the output after feature fusion, and the output after feature fusion and full connection to understand their action states. We observed that the raw data without any processing were highly mixed and challenging to cluster, whether it was t-SNE or PCA clustering. After feature fusion, the data after t-SNE clustering were mostly clustered together, but more clusters were formed after clustering, and the data after PCA clustering were clearly divided into two categories. The cluster output after full connection showed a clear cluster diagram through t-SNE and PCA clustering, with only a few misclassified samples. Therefore, our MLACNN network can effectively extract and abstract the high-level features of the original methylation data to better classify the methylation data.

## Challenges and future work

Although the MLACNN model performs reasonably well in predicting methylation sites, there is still significant room for improvement. With ample computing power, we can adopt strategies such as automatic parameter search or automatic kernel search to enhance the proposed network architecture, for example, using Efficientnet (Tan and Le 2019) and NASNet (Qin and Wang 2019). Additionally, we can leverage the parallel feature extraction strategy of multi-convolution, similar to GoogleNet (Van der Maaten and Hinton 2008), to learn more features at each layer of the neural network. If we plan to deploy our model on mobile devices, such as mobile phones, we may need to compress the network parameters. We can use techniques such as separable convolution to reduce the parameters of our convolution kernel and minimize the model size.

## Conclusion

In this paper, we propose a multi-latitude attention convolutional neural network for accurately predicting the methylation sites of m5C. Our approach uses an attention-based feature fusion method to fuse and predict the features extracted from the NCP network, one-hot network, and EIIP-vector network. Each network is a bottleneck with channel shuffle based on attention mechanism, and employs one-dimensional convolution and *D*-dimensional convolution for feature extraction while preventing overfitting through max pooling, early stop, batch normalization, dropout, and *L*1 and *L*2 regularization. We compared the models obtained by experiments of NCP network, one-hot network and EIIP-vector network with the models after feature fusion to obtain the best fusion model. Additionally, we performed t-SNE and PCA clustering analysis on the original methylation data, the methylation data after feature fusion, and the methylation data after full connection to analyze the working principle of each layer of the neural network.

## Data Availability

MLACNN is available as open source Python software (https://github.com/jrebai/MLACNN.), The raw geo and grch37 datasets are available from https://www.ncbi.nlm.nih.gov/geo/query/acc.cgi?acc=GSM432685, https://www.ncbi.nlm.nih.gov/geo/query/acc.cgi?acc=GSM2868190, https://www.ncbi.nlm.nih.gov/geo/query/acc.cgi?acc=GSM2868192, https://www.ncbi.nlm.nih.gov/geo/query/acc.cgi?acc=GSM2868191, and https://www.ncbi.nlm.nih.gov/geo/query/acc.cgi?acc=GSM2868193. ftp://ftp.ncbi.nlm.nih.gov/genomes/archive/old_genbank/Eukaryotes/vertebrates_mammals/Homo_sapiens/GRCh37/Primary_Assembly/assembled_chromosomes/FASTA/
